# Whole-genome sequencing reveals lineage-associated drug resistance and enables machine learning–based prediction in *Mycobacterium tuberculosis* clinical isolates

**DOI:** 10.3389/fmicb.2026.1828991

**Published:** 2026-07-22

**Authors:** Zelin Hao, Chao Wu, Yi Peng, Lijun Zhou, Jinyao Li, Xiaoguang Zou

**Affiliations:** 1 College of Life Science and Technology, Xinjiang University, Urumqi, China; 2 Department of Infectious Diseases, First People′s Hospital of Kashi, Kashi, China; 3 The Sixth People's Hospital of Xinjiang Uygur Autonomous Region, Urumqi, China; 4 School of Basic Medical Sciences, Xinjiang Medical University, Urumqi, China

**Keywords:** antimicrobial resistance, drug-resistant tuberculosis, genomic epidemiology, machine learning, *Mycobacterium tuberculosis*, whole-genome sequencing

## Abstract

**Background:**

Drug-resistant tuberculosis (TB) remains a major obstacle to global TB control, particularly in high-burden settings where timely detection of resistance is limited. This study aimed to integrate whole-genome sequencing and machine learning approaches to characterize the genomic architecture of drug resistance and develop predictive models for phenotypic resistance in clinical *Mycobacterium tuberculosis* isolates from Kashi, Xinjiang, China.

**Methods:**

A total of 160 clinical *M. tuberculosis* isolates were collected from culture-confirmed pulmonary tuberculosis patients at First People’s Hospital of Kashi over a five-year period (January 2020–December 2024). Whole-genome sequencing was performed, and sequencing reads were mapped to the H37Rv reference genome for high-confidence single nucleotide polymorphism (SNP) and insertion/deletion (INDEL) calling. Resistance-associated mutations were identified using curated resistance mutation catalogues, and comparative genomic analyses were conducted to assess mutation burden across phenotypic resistance groups. Phylogenetic reconstruction based on genome-wide SNPs was performed to evaluate lineage distribution and clustering of resistant strains. In addition, random forest machine learning models were trained using WGS-derived coding and promoter mutations to predict phenotypic resistance to first-line anti-tuberculosis drugs.

**Results:**

Phenotypic drug susceptibility testing classified 63 isolates (39.4%) as drug-sensitive, 67 (41.9%) as single-drug resistant (SDR), and 28 (17.5%) as multidrug-resistant (MDR), with a small number of extensively drug-resistant isolates. Resistance was dominated by first-line drugs, particularly isoniazid, streptomycin, and rifampicin. Phylogenetic analysis revealed non-random distribution of resistant isolates, with enrichment of MDR strains within Lineage 2 (Beijing lineage). Genomic profiling demonstrated a progressive increase in resistance-associated mutational burden from drug-sensitive to SDR and MDR isolates, characterized by accumulation of canonical mutations in *rpo*B, *kat*G, *emb*B, *gyr*A, *pnc*A, and *rrs*. Machine learning–based prediction achieved strong performance for rifampicin, isoniazid, and streptomycin resistance, with key resistance-conferring mutations and promoter variants emerging as dominant predictive features.

**Conclusion:**

This study demonstrates the potential utility of integrating phenotypic testing, whole-genome sequencing, and interpretable machine learning approaches for characterizing and predicting drug-resistant *M. tuberculosis*. The observed burden of resistance-associated mutations and lineage-associated phylogenetic clustering of resistant isolates support the value of WGS-based surveillance and predictive modelling for tuberculosis control strategies.

## Introduction

Tuberculosis (TB) remains one of the world’s deadliest infectious diseases, with an estimated 10.8 million people falling ill in 2023, equivalent to 134 cases per 100,000 population, following a rebound in incidence after COVID-19-related disruptions to TB services (Goletti et al., 2025). Although slightly higher than 2022 estimates, this figure highlights that global progress toward TB elimination has stalled and the disease continues to impose enormous health burdens worldwide (Suvvari, 2025). The majority of incident TB cases occur in the WHO regions of South-East Asia (≈45%), Africa (≈24%) and the Western Pacific (≈17%), underscoring stark geographic disparities in disease burden (Xie et al., 2025; World Health Organization, 2024). Drug-resistant TB in particular multidrug-resistant (MDR) or rifampicin-resistant TB (RR-TB) compounds the challenge of achieving effective control (Kurz et al., 2016). WHO estimates suggest that ≈400,000 people developed MDR/RR-TB in 2023, with India alone accounting for roughly 27% of global MDR/RR-TB incident cases, followed by the Russian Federation (7.4%), Indonesia (7.4%), China (7.3%) and the Philippines (7.2%). The estimated 150,000 deaths attributable to MDR/RR-TB in 2023 reflect persistent mortality associated with drug resistance and emphasize the critical need for timely detection and intervention (Global Tuberculosis Report 2024, 2024). Despite the longstanding global TB control agenda and widespread use of anti-TB chemotherapy, conventional phenotypic drug susceptibility testing (pDST) remains limited by intrinsic constraints (Yusoof et al., 2022). Phenotypic approaches require culture of this slow-growing organism and often take weeks to yield results, delaying initiation of appropriate therapy. Technical variability across laboratories and the inability to discriminate mixed infections or resolve the genetic basis of resistance further limit the utility of pDST in clinical decision-making and epidemiologic surveillance. These issues are particularly acute for MDR/RR-TB and other complicated resistance patterns where rapid, accurate detection is essential to guide effective treatment and limit transmission (Zaporojan et al., 2025). Whole-genome sequencing (WGS) of *M. tuberculosis* has emerged as a transformative tool that can overcome many of these limitations (Advani et al., 2019). WGS enables comprehensive detection of known resistance-conferring mutations across the entire bacterial genome, offers high resolution for strain differentiation, and when integrated with phylogenetic analyses facilitates reconstruction of transmission networks and lineage distributions. As sequencing platforms become more accessible and bioinformatics workflows more standardized, WGS is increasingly being deployed in surveillance systems and clinical settings, providing faster and more informative data than conventional methods. In this study, we applied whole-genome sequencing to culture-confirmed *M. tuberculosis* isolates collected in Kashi to comprehensively characterize genomic diversity, lineage structure, and resistance-associated mutational profiles in a real-world clinical setting. By integrating phenotypic drug susceptibility testing, phylogenetic reconstruction, and machine learning–based resistance prediction, we examined how resistance-conferring mutations accumulate across phenotypic resistance categories and cluster within specific genetic lineages among treatment-naïve patients. Our objective was to evaluate the added value of WGS for accurate resistance detection, population-level surveillance, and epidemiological insight, thereby informing timelier and precision-guided tuberculosis care in a high-burden urban setting.

## Materials and methods

### Study design and patient population

This study was conducted as a retrospective observational cohort study between January 2020 and December 2024, involving patients with culture-confirmed pulmonary TB. Clinical, microbiological, and genomic data were retrieved from hospital medical records and laboratory information systems over the defined study period. Patients were eligible for inclusion if they had microbiologically confirmed *M. tuberculosis* infection based on positive culture, availability of respiratory specimens suitable for whole-genome sequencing (WGS), and complete demographic and clinical information and treatment outcomes. Patients were excluded if they had non-tuberculous mycobacterial infections, culture-negative TB, or incomplete clinical or outcome data. To avoid duplication and bias related to repeated sampling, only the first isolate per patient was included in the analysis. The following clinical variables were collected: age, sex, and treatment outcomes, categorized as cure, treatment failure, relapse, and death. Age was analysed both as a continuous variable and stratified into clinically relevant categories for subgroup analyses.

### Sample collection and culture

Respiratory specimens, including sputum and bronchoalveolar lavage (BAL) samples, were collected from enrolled patients under biosafety level 2 and 3 conditions, in accordance with institutional infection control protocols. A total of 160 respiratory specimens were included in the final analysis, corresponding to one isolate per patient. Specimens were processed using standard N-acetyl-L-cysteine–sodium hydroxide (NALC–NaOH) decontamination procedures, followed by concentration through centrifugation. Processed samples were inoculated in parallel into liquid culture using the Mycobacteria Growth Indicator Tube (MGIT) 960 system and onto solid Löwenstein–Jensen (LJ) medium to maximize recovery of *M. tuberculosis* (Cruciani et al., 2004). MGIT cultures were incubated at 37 °C and monitored automatically for growth for up to 42 days, while LJ cultures were incubated at 37 °C and inspected weekly for a maximum of 8 weeks. Culture positivity was confirmed by acid-fast bacilli staining and molecular assays. Species identification of culture-positive isolates was performed using standard biochemical tests and molecular methods, in accordance with national tuberculosis laboratory guidelines. Only isolates confirmed as *M. tuberculosis* complex were included for downstream whole-genome sequencing.

### Whole-genome DNA extraction and sequencing

Genomic DNA was extracted from 160 non-duplicated, culture-positive *M. tuberculosis* isolates, with one isolate per patient included in the analysis (Belisle et al., 2009). Bacterial biomass was harvested from 5 mL liquid cultures and subjected to DNA extraction using either a cetyltrimethylammonium bromide (CTAB)–based protocol optimized for disruption of the lipid-rich mycobacterial cell wall, or the Qiagen DNeasy UltraClean Microbial Kit (Qiagen, Germany), following the manufacturer’s instructions. For the CTAB-based method, bacterial pellets were heat-inactivated at 80 °C for 30 min to ensure biosafety, followed by mechanical lysis through bead beating and chemical lysis using lysozyme and proteinase-K in the presence of sodium dodecyl sulfate (SDS). DNA was subsequently purified by phenol–chloroform extraction and ethanol precipitation, and resuspended in nuclease-free buffer. DNA quantity and purity were assessed using NanoDrop spectrophotometry, evaluating A260/280 and A260/230 ratios, and Qubit dsDNA fluorometric assays (Thermo Fisher Scientific) for accurate concentration measurement. Only DNA samples with A260/280 ratios between 1.8 and 2.0, acceptable A260/230 ratios, and sufficient yield for library preparation were advanced to whole-genome sequencing. Whole-genome sequencing was performed on an Illumina sequencing platform (e.g., Illumina NextSeq 550) using paired-end 2 × 150 bp chemistry (Quail et al., 2012). Sequencing libraries were constructed from 1 ng of input genomic DNA using the Illumina Nextera XT DNA Library Preparation Kit (Illumina, United States), which employs enzymatic tagmentation for simultaneous DNA fragmentation and adapter ligation. Unique dual indices were incorporated during limited-cycle PCR amplification to enable multiplex sequencing. Following library preparation, fragment size distribution was assessed using capillary electrophoresis, and libraries with an average insert size of approximately 300–600 bp were retained. Libraries were quantified fluorometrically, normalized to equimolar concentrations, and pooled prior to sequencing. Cluster generation and sequencing were performed according to the manufacturer’s standard workflow using Illumina sequencing-by-synthesis chemistry. Sequencing depth was designed to achieve a minimum mean genome coverage of ≥50 × across the ~4.4 Mb *M. tuberculosis* genome, a threshold selected to ensure high-confidence detection of SNPs and small insertions/deletions (INDELs) while minimizing false-positive variant calls in repetitive genomic regions. The mean sequencing depth achieved across all isolates was 274.20 (median: 273.09, with a range of 44.36–372.35, confirming consistent and sufficient coverage for reliable variant detection). Run-level quality metrics, including cluster density, percentage of bases with Q scores ≥30, and read duplication rates, were monitored to ensure sequencing performance prior to downstream bioinformatic analysis. Raw sequencing reads were first assessed using FastQC v0.11.9 and processed with Trimmomatic v0.39 to remove adapter sequences, trim low-quality bases (Phred score <20), and discard reads shorter than 50 bp after trimming. Duplicate read rates were evaluated prior to variant calling; however, duplicate removal was not performed because low duplication levels were observed across samples. High-quality reads were aligned to the *M. tuberculosis* H37Rv reference genome (NC_000962.3) using BWA-MEM v0.7.17. Variant calling was performed using SAMtools mpileup and BCFtools v1.17. Variants were retained only if they met the following high-confidence criteria: minimum read depth ≥10, mapping quality ≥30, base quality ≥20, and alternate allele frequency ≥75%. To minimize false-positive calls, repetitive genomic regions, PE/PPE gene families, insertion sequence elements, and other low-complexity regions were excluded from downstream analyses. The mean mapping rate was 96%, and the average genome coverage breadth was 98% across all isolates. Samples with genome coverage breadth <95% or evidence of contamination >5% were excluded from analysis. Lineage assignment was performed using TB-Profiler (v6.4.0) based on established lineage-defining single nucleotide polymorphism (SNP) barcodes. Genomic features used for machine learning included high-confidence non-synonymous mutations and promoter variants within established resistance-associated loci, guided by WHO-endorsed resistance mutation catalogues and relevant literature, while synonymous and low-confidence variants were excluded. High-quality SNPs identified from whole-genome sequencing data were used to construct a phylogenetic tree. A core genome SNP alignment was generated after excluding repetitive and low-confidence regions. Maximum-likelihood phylogenetic analysis was performed using IQ-TREE v2.2 under the general time-reversible model with ascertainment bias correction, and branch support was assessed using 1,000 bootstrap replicates. The resulting tree was visualized and annotated using R ggtree, incorporating lineage classification, phenotypic resistance profiles, and genome quality metrics.

### Drug resistance prediction

Genotypic drug resistance was predicted from whole-genome sequencing data based on well-established resistance-associated mutations in *M. tuberculosis.* For rifampicin resistance, mutations within the rifampicin resistance–determining region (RRDR) of *rpo*B were prioritized, including canonical substitutions such as S450L, H445Y/D, and D435V. Isoniazid resistance was inferred from mutations in *kat*G (S315T) and nucleotide substitutions in the *inh*A promoter region (15C > T), reflecting high-level and low-level resistance mechanisms, respectively. Fluoroquinolone resistance was assessed through mutations in the quinolone resistance–determining regions (QRDRs) of *gyr*A (e.g., A90V, S91P, D94G/A/N) and *gyr*B (N538D, E540V). Ethambutol resistance was evaluated using mutations in *emb*B, particularly M306V/I, while streptomycin resistance was inferred from substitutions in *rps*L (K43R, K88R) and *rrs* (A514C, C517T). Resistance annotation was performed using curated *M. tuberculosis* resistance mutation catalogues aligned with WHO-endorsed classification frameworks, incorporating only high-confidence mutations with robust experimental or epidemiological evidence for resistance. Isolates harbouring resistance-conferring mutations to at least rifampicin and isoniazid were classified as multidrug-resistant TB (MDR-TB), while additional resistance to fluoroquinolones and/or second-line injectable agents was used to define pre-XDR or XDR-TB, as applicable.

### Disputed and low-confidence mutations

Mutations with inconsistent or limited evidence for resistance association were classified separately as disputed or low-confidence mutations. These included rare non-synonymous substitutions or promoter variants reported in the literature with variable phenotypic impact. Such mutations were not used alone to assign resistance status, but were retained for descriptive and exploratory genomic analyses to avoid overestimation of resistance prevalence. The present study focused primarily on resistance profiling, phylogenetic characterization, and machine learning–based resistance prediction using WGS-derived features. Comprehensive genotype–phenotype concordance analyses were added with detailed phenotypic validation data.

### Statistical analysis

Statistical analyses were performed to assess associations between genomic resistance-associated mutations, phenotypic drug resistance, and clinical outcomes. Categorical variables were compared using chi-square or Fisher’s exact tests, while continuous variables were analyzed using Student’s t-test or Mann–Whitney U test, as appropriate. Multivariable logistic regression was used to identify independent predictors of drug resistance and adverse outcomes, adjusting for age, sex, and *M. tuberculosis* lineage. Results were expressed as adjusted odds ratios (aORs) with 95% confidence intervals (CIs). All tests were two-sided, and a *p*-value <0.05 was considered statistically significant. Analyses were conducted using R and IBM SPSS Statistics software. Random forest classifiers were implemented using the randomForest package in R. The number of trees was set to 500, and the number of features considered at each split was determined as the square root of the total number of input features. Hyperparameter tuning was performed using cross-validation to optimize model performance and reduce overfitting. Stratified 5-fold cross-validation was used to ensure balanced representation of resistant and susceptible isolates during model training and evaluation.

### Ethical approval

This study was conducted in accordance with the ethical principles outlined in the Declaration of Helsinki. Ethical approval was obtained from the Institutional Review Board of the First People’s Hospital of Kashi (IRB approval number: [2024] Quick Review Research No. 30). Given the retrospective nature of the study and the use of anonymized data, the requirement for written informed consent was waived by the IRB.

## Results

### Study cohort and isolate characteristics

A total of 160 culture-confirmed *M. tuberculosis* isolates, corresponding to 160 individual patients, were included in the final analysis, with only the first isolate per patient considered to avoid duplication and sampling bias. The annual distribution of isolates was as follows: *n* = 28 (17.5%) in 2020, *n* = 31 (19.4%) in 2021, *n* = 34 (21.3%) in 2022, *n* = 33 (20.6%) in 2023, and *n* = 34 (21.3%) in 2024. All isolates were obtained from respiratory specimens, including sputum and bronchoalveolar lavage samples, and fulfilled the predefined inclusion criteria for downstream genomic and drug resistance analyses. The study cohort comprised 90 male (56.3%) and 70 female (43.8%) patients, indicating a male predominance consistent with the epidemiology of pulmonary tuberculosis. The mean age of patients was 59.0 ± 17.3 years (range: 13–92 years). For subgroup analyses, age was further stratified into clinically relevant categories, with the majority of patients falling within the ≥ 60-year age group (42.5%), followed by 40–59 years (35.6%), 20–39 years (16.3%), and < 20 years (5.6%). All patients had microbiologically confirmed pulmonary tuberculosis based on positive culture, and complete demographic information was available for all included cases. The availability of high-quality respiratory isolates enabled robust phenotypic resistance profiling and ensured suitability for whole-genome sequencing–based analyses.

### Phenotypic drug resistance distribution in the study cohort

**Figure 1:**
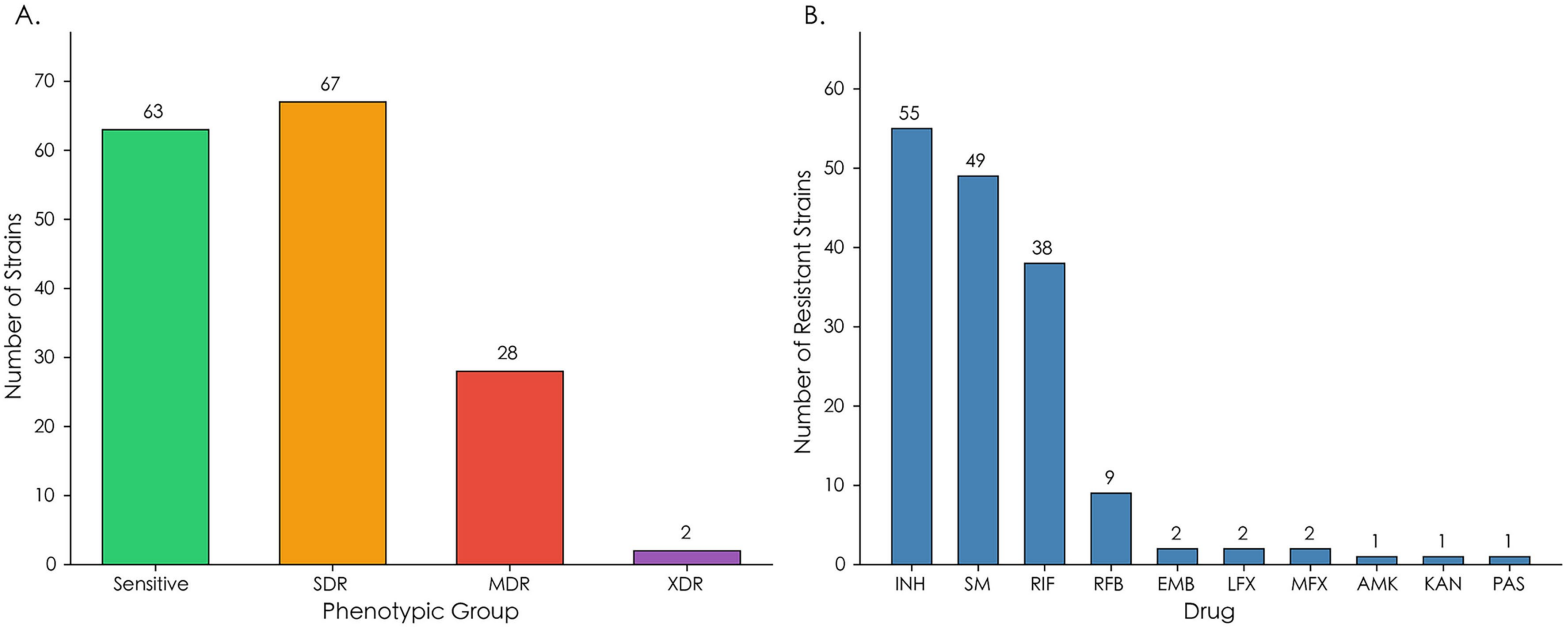
Phenotypic drug resistance distribution among *Mycobacterium tuberculosis* clinical isolates (*N* = 160). **(A)** Distribution of *M. tuberculosis* isolates according to phenotypic drug resistance categories. SDR: Single-drug resistance (SDR); MDR n: 28 Multidrug-resistant; XDR: Extensively drug-resistant tuberculosis **(B)** Drug-wise distribution of phenotypic resistance across the study cohort. The number of isolates resistant to each anti-tuberculosis drug is shown, with isoniazid (INH) resistance being most prevalent, followed by streptomycin (SM) and rifampicin (RIF). Resistance to rifabutin (RFB), ethambutol (EMB), fluoroquinolones (levofloxacin [LFX] and moxifloxacin [MFX]), second-line injectable agents (amikacin [AMK] and kanamycin [KAN]), and para-aminosalicylic acid (PAS) was infrequent.

A total of 160 non-duplicated *M. tuberculosis* isolates were included in the final phenotypic and genomic analyses. Based on phenotypic drug susceptibility testing, isolates were classified into four resistance categories (Figure 1A). Of these, *n* = 63 (39.4%) was fully drug-sensitive, *n* = 67 (41.9%) exhibited single-drug resistance (SDR), 28 (17.5%) were multidrug-resistant (MDR), and *n* = 2 (1.3%) was extensively drug-resistant (XDR). Resistance to isoniazid (INH) was most frequent (55/160, 34.4%), followed by streptomycin (SM; 49/160, 30.6%) and rifampicin (RIF; 38/160, 23.8%). Resistance to rifabutin (RFB) was detected in *n* = 9 isolates (5.6%), while resistance to ethambutol (EMB), fluoroquinolones (levofloxacin and moxifloxacin), and second-line injectable agents was rare (Figure 1B) These results indicate that first-line drug resistance predominates in the study population.

### Phylogenetic structure and lineage-specific distribution of resistance

**Figure 2:**
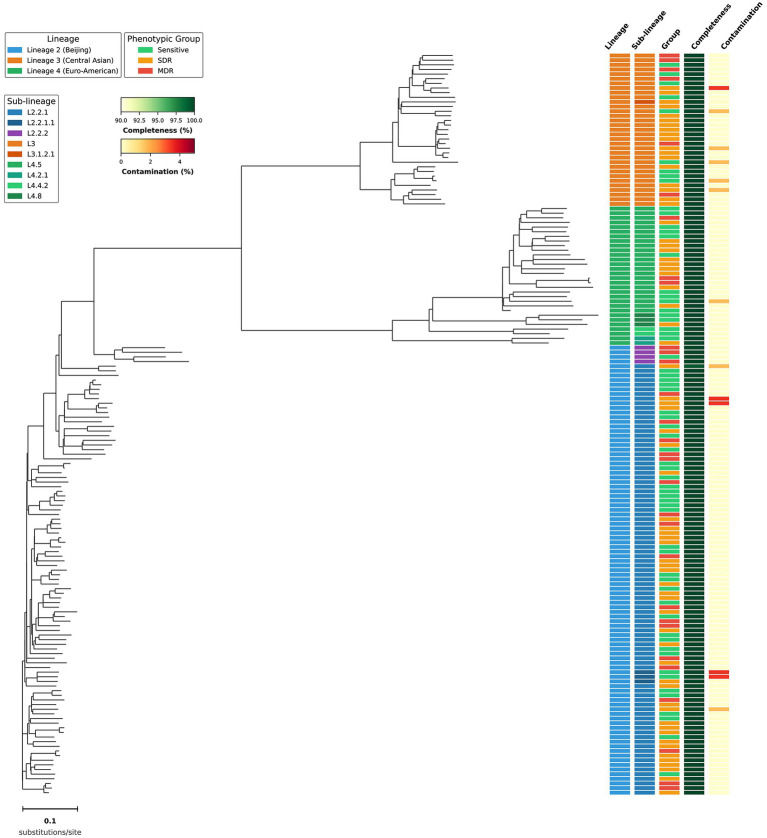
Whole-genome SNP–based phylogenetic analysis and lineage-associated distribution of drug resistance in *Mycobacterium tuberculosis*. A maximum-likelihood phylogenetic tree constructed from high-confidence genome-wide single nucleotide polymorphisms (SNPs) is shown. Isolates are annotated by major lineage and sub-lineage (left color bars), including Lineage 2 (Beijing), Lineage 3 (Central Asian), and Lineage 4 (Euro-American). Phenotypic drug resistance categories (drug-sensitive, single-drug resistant [SDR], multidrug-resistant [MDR]) are overlaid alongside the tree. Additional annotation tracks depict genome assembly completeness (%) and contamination levels (%) for each isolate. The scale bar indicates substitutions per site. MDR isolates are predominantly clustered within Lineage 2, whereas drug-sensitive isolates are distributed across multiple lineages.

Whole-genome SNP–based phylogenetic reconstruction revealed clear clustering of isolates into the major *M. tuberculosis* lineages (Figure 2). The majority of isolates belonged to Lineage 2 (Beijing lineage), followed by Lineage 3 (Central Asian lineage) and Lineage 4 (Euro-American lineage). Sub-lineage annotation further demonstrated genetic diversity within each lineage. Overlay of phenotypic resistance categories onto the phylogenetic tree demonstrated that MDR and XDR isolates were non-randomly distributed, with enrichment observed predominantly within Lineage 2 clusters (Figure 2, phenotype annotation panel). Both XDR isolates clustered within Lineage 2 (Beijing lineage). In contrast, drug-sensitive isolates were distributed across all major lineages. Genome assembly quality metrics, including high completeness (>95%) and low contamination, were consistent across lineages, indicating robust sequencing quality (Figure 2, quality annotation panel).

### Genomic distribution of resistance-associated mutations across phenotypic groups

**Figure 3:**
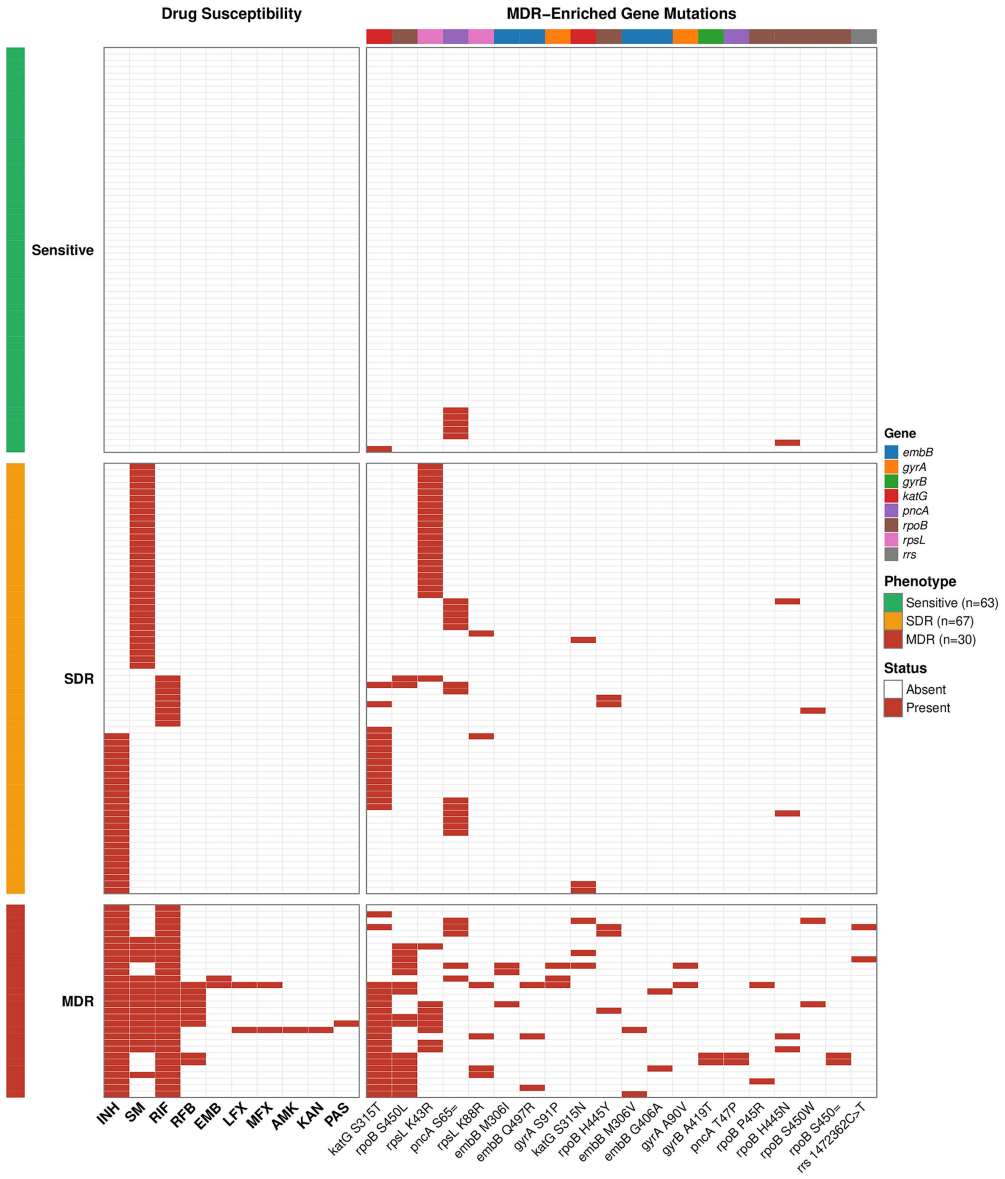
Distribution of resistance-associated mutations across phenotypic groups of *Mycobacterium tuberculosis* isolates. SDR: Single-drug resistant; MDR: Multidrug-resistant. Columns represent resistance-associated genes and specific mutations linked to first-line and second-line anti-tuberculosis drugs, while rows represent individual isolates. Coloured blocks indicate the presence of a mutation, whereas absence is shown in white. Canonical resistance mutations in katG, rpoB, rpsL, embB, gyrA, gyrB, pncA, and rrs are enriched in MDR isolates, while SDR isolates predominantly harbour single resistance-conferring mutations.

To investigate the relationship between phenotypic drug resistance and underlying genomic determinants, resistance-associated mutations were systematically mapped across drug-sensitive, SDR, and MDR *M. tuberculosis* isolates. Drug-sensitive isolates were largely devoid of canonical resistance-conferring mutations across major resistance-associated loci, consistent with their phenotypic susceptibility profiles (Figure 3). In contrast, SDR isolates typically harboured single high-confidence resistance mutations, reflecting mono-resistance patterns. The most frequently observed mutations in SDR isolates included *kat*G S315T (isoniazid resistance), *rps*L K43R or K88R (streptomycin resistance), and substitutions within the rifampicin resistance–determining region (RRDR) of *rpo*B, in agreement with phenotypic drug susceptibility testing (Figure 3; Supplementary Figure S1). MDR isolates demonstrated a markedly different mutational landscape, characterized by the accumulation of multiple resistance-conferring mutations across distinct drug classes (Figure 3). Prominent mutations included *rpo*B S450L and H445Y (rifampicin resistance), *kat*G S315T and S315N (isoniazid resistance), *emb*B M306V/I (ethambutol resistance), and *gyr*A quinolone resistance–determining region mutations (A90V, S91P), consistent with combined resistance to first-line and second-line agents. Additional mutations in genes such as *pnc*A, *gyr*B, and *rrs* were observed in subsets of MDR isolates, suggesting further resistance amplification and diversification. The progressive increase in both the number and diversity of resistance-associated mutations from SDR to MDR isolates supports a stepwise evolutionary trajectory of drug resistance acquisition, driven by sequential antimicrobial selective pressures. These genomic patterns provide mechanistic support for the observed phenotypic resistance profiles and underscore the value of whole-genome sequencing for resolving complex resistance architectures in tuberculosis. To further characterize the genomic basis of resistance, the distribution of major resistance-associated mutations was compared across phenotypic resistance groups (Table 1). Canonical resistance determinants, including mutations in *rpo*B, *kat*G, *emb*B, *rps*L, and *gyr*A, were progressively enriched from sensitive to SDR and MDR isolates, supporting the observed stepwise accumulation of resistance-associated genomic alterations.

**Table 1:** Distribution of resistance-associated mutations across phenotypic resistance groups.

Resistance determinant	Sensitive (*n* = 63)	SDR (*n* = 67)	MDR/XDR (*n* = 30)	Total positive (%)
*rpo*B mutations	1/63 (1.6%)	12/67 (17.9%)	27/30 (90.0%)	40/160 (25.0%)
*kat*G mutations	2/63 (3.2%)	21/67 (31.3%)	23/30 (76.7%)	46/160 (28.7%)
*inh*A promoter mutations	0/63 (0.0%)	3/67 (4.5%)	2/30 (6.7%)	5/160 (3.1%)
*em*bA/*emb*B mutations	0/63 (0.0%)	3/67 (4.5%)	13/30 (43.3%)	16/160 (10.0%)
*rps*L mutations	0/63 (0.0%)	24/67 (35.8%)	12/30 (40.0%)	36/160 (22.5%)
*rrs* mutations	1/63 (1.6%)	2/67 (3.0%)	4/30 (13.3%)	7/160 (4.4%)
*gyr*A mutations	0/63 (0.0%)	2/67 (3.0%)	5/30 (16.7%)	7/160 (4.4%)
*gyr*B mutations	0/63 (0.0%)	0/67 (0.0%)	1/30 (3.3%)	1/160 (0.6%)
*pnc*A mutations	0/63 (0.0%)	0/67 (0.0%)	9/30 (30.0%)	9/160 (5.6%)

### Machine learning–based prediction of drug resistance and key genomic determinants

**Figure 4:**
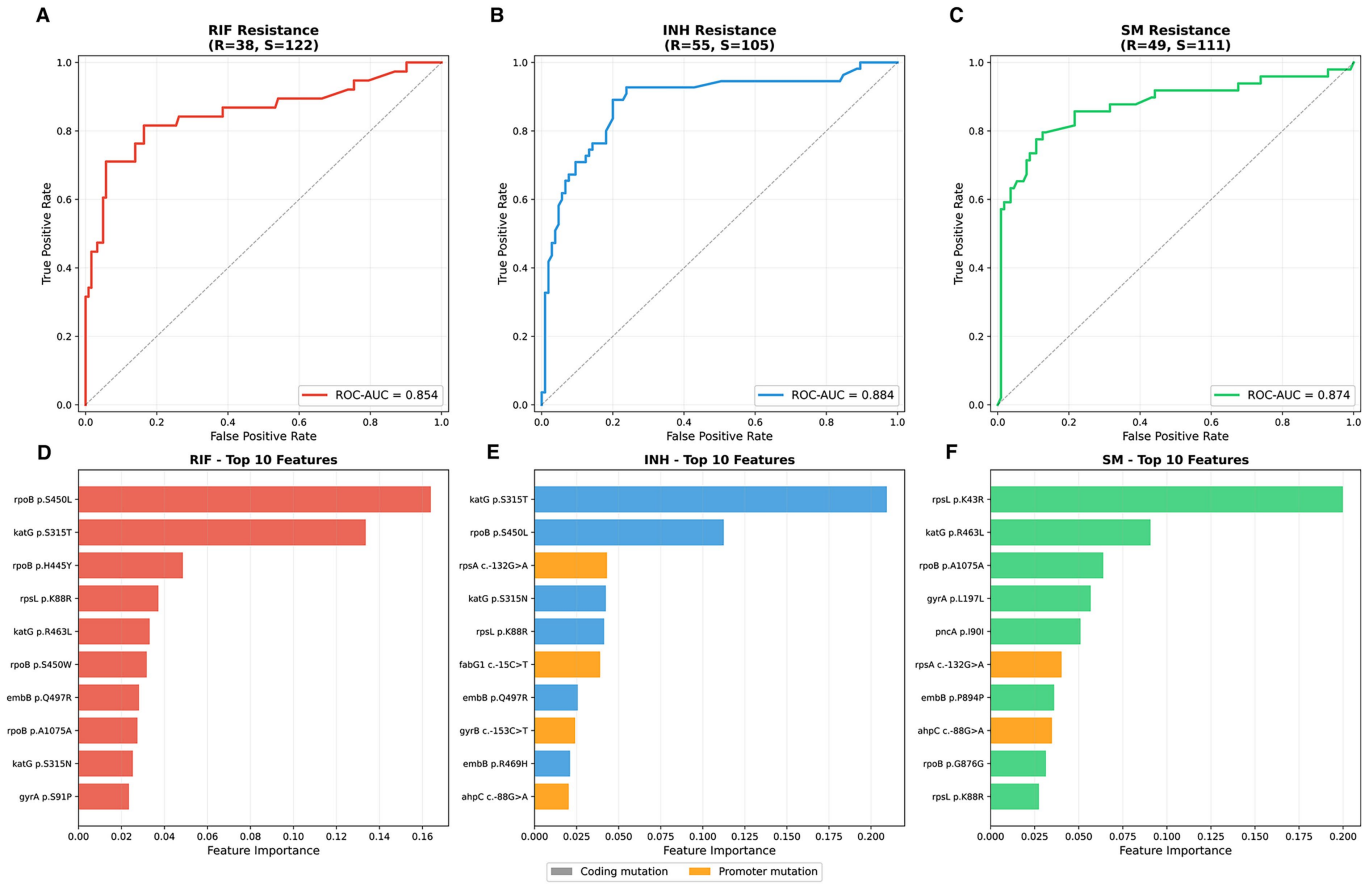
Machine learning–based prediction of anti-tuberculosis drug resistance using whole-genome sequencing features. **(A)** Receiver operating characteristic (ROC) curve showing the performance of the random forest classifier for rifampicin (RIF) resistance prediction. The model was trained using whole-genome sequencing–derived features and evaluated by 5-fold stratified cross-validation across 160 *M. tuberculosis* isolates (38 resistant, 122 susceptible). The area under the ROC curve (ROC–AUC) indicates strong discriminative performance. **(B)** ROC curve illustrating classifier performance for isoniazid (INH) resistance prediction (55 resistant, 105 susceptible isolates). The model demonstrates high predictive accuracy, reflecting robust genomic signal capture for isoniazid resistance. **(C)** ROC curve for streptomycin (SM) resistance prediction (49 resistant, 111 susceptible isolates), showing strong classification performance comparable to rifampicin and isoniazid models. **(D)** Top 10 genomic features ranked by importance for rifampicin resistance prediction. Canonical mutations within the rpoB rifampicin resistance–determining region (RRDR), including rpoB S450L and H445Y, are the most influential predictors. **(E)** Top 10 genomic features contributing to isoniazid resistance prediction, dominated by katG S315T, with additional substantial contributions from inhA promoter mutations, highlighting the combined impact of coding and regulatory variants. **(F)** Top 10 genomic features driving streptomycin resistance prediction, with rpsL K43R as the strongest predictor, consistent with established mechanisms of high-level streptomycin resistance. **(D–F)** Coding mutations and promoter mutations are distinguished, demonstrating that inclusion of regulatory variants enhances model interpretability and predictive performance.

To evaluate the predictive utility of WGS-derived features for phenotypic drug resistance, random forest classifiers were trained using 252 genomic features, comprising both coding mutations and promoter variants across established antibiotic resistance–associated loci. Model performance was assessed using 5-fold stratified cross-validation on the full dataset of 160 *M. tuberculosis* isolates, ensuring balanced representation of resistant and susceptible strains for each drug class (Figure 4). Receiver operating characteristic (ROC) curve analysis demonstrated strong discriminative performance for prediction of resistance to first-line anti-tuberculosis drugs (Figures 4A–C). Specifically, the classifier achieved a ROC–AUC of 0.854 for rifampicin resistance (38 resistant vs. 122 susceptible isolates; Figure 4A), a ROC–AUC of 0.884 for isoniazid resistance (55 resistant vs. 105 susceptible isolates; Figure 4B), and a ROC–AUC of 0.874 for streptomycin resistance (49 resistant vs. 111 susceptible isolates; Figure 4C). In addition, model performance was further evaluated using sensitivity, specificity, and overall accuracy at the optimal classification threshold (determined by the Youden index). For rifampicin, isoniazid, and streptomycin resistance prediction, the models achieved sensitivity of 73.7, 89.1, and 79.6%, specificity of 91.8, 80.0, and 89.2%, and overall accuracy of 87.5, 83.1, and 86.2%, respectively. Additional performance metrics further supported the predictive utility of the models. At the optimal classification threshold determined by the Youden index, the corresponding positive predictive values (PPVs), negative predictive values (NPVs), and 95% confidence intervals are summarized in Supplementary Table S1. These findings provide additional evidence of the clinical applicability and robustness of the WGS-based resistance prediction framework. These results indicate robust predictive accuracy across drug classes despite class imbalance, underscoring the capacity of WGS-derived features to capture key resistance determinants. To identify the genomic features contributing most strongly to resistance prediction, feature importance scores were extracted from the trained random forest models (Figures 4D–F). Across all three drug classes, canonical resistance-conferring mutations emerged as the dominant predictors, providing biological validation of the machine learning framework. For rifampicin resistance, mutations within the rifampicin resistance–determining region (RRDR) of *rpoB* were the most influential features, with *rpoB* S450L and *rpoB* H445Y exhibiting the highest importance scores (Figure 4D). Additional contributions from other *rpoB* substitutions and mutations in associated loci further supported the central role of RNA polymerase alterations in rifampicin resistance. Prediction of isoniazid resistance was driven primarily by *katG* S315T, consistent with its established association with high-level isoniazid resistance (Figure 4E). Notably, *inhA* promoter mutations (–15C > T) also ranked among the top predictors, highlighting the contribution of regulatory variants to resistance phenotypes. The combined importance of coding and promoter mutations underscores the multifactorial genetic basis of isoniazid resistance. For streptomycin resistance, the strongest predictor was *rpsL* K43R, a well-characterized mutation conferring high-level resistance (Figure 4F). Additional contributions from mutations in *katG*, *rpoB*, and other loci suggest potential linkage effects or compensatory adaptations captured by the model. Importantly, promoter mutations consistently contributed to predictive performance, particularly for isoniazid resistance, demonstrating that inclusion of non-coding regulatory variation enhances resistance prediction accuracy beyond coding mutations alone. Collectively, these findings confirm that the random forest models not only achieve promising predictive performance but also recapitulate known biological mechanisms of drug resistance while identifying additional contributory genomic signals. Together, Figures 4A–F demonstrates that machine learning models trained on comprehensive WGS-derived mutation profiles can accurately predict phenotypic drug resistance, while maintaining interpretability through biologically meaningful feature importance rankings. The concordance between top-ranked features and established resistance mechanisms supports the validity of this approach for genomic resistance surveillance and provides a framework for extending prediction to less-characterized drugs and resistance pathways.

### Integrated interpretation of phenotypic, phylogenetic, and predictive analyses

The combined phenotypic, phylogenetic, and genomic analyses reveal a coherent and biologically consistent landscape of drug resistance in the studied *M. tuberculosis* population. Phenotypic drug susceptibility testing demonstrated a substantial burden of first-line drug resistance, with a high frequency of single-drug resistance and a notable proportion of multidrug-resistant isolates, while extensively drug-resistant strains remained uncommon. Resistance was dominated by isoniazid, streptomycin, and rifampicin, indicating sustained selective pressure from standard treatment regimens. Whole-genome phylogenetic analysis showed that resistant isolates were not randomly distributed but were enriched within specific genetic backgrounds, particularly Lineage 2, whereas drug-sensitive isolates were distributed across multiple lineages, suggesting lineage-associated phylogenetic clustering of resistant isolates within specific genetic backgrounds, together with lineage-associated fitness advantages, may contribute to resistance dissemination. Genomic profiling of resistance-associated mutations revealed a progressive increase in mutational burden from drug-sensitive to single-drug resistant and ultimately multidrug-resistant isolates, characterized by the stepwise accumulation of high-confidence mutations across multiple resistance-associated loci, providing molecular evidence for sequential resistance acquisition under antimicrobial pressure. Machine learning–based resistance prediction using whole-genome sequencing features achieved promising predictive performance and consistently identified canonical resistance mutations as dominant contributors, while also highlighting the substantial role of promoter and other regulatory variants in shaping resistance phenotypes. These findings support the potential utility of integrating whole-genome sequencing and interpretable machine learning approaches for tuberculosis drug resistance surveillance and precision diagnostics.

## Discussion

In this study, we integrated phenotypic drug susceptibility testing, whole-genome sequencing based phylogenetics, resistance mutation profiling, and machine learning based prediction to comprehensively characterize drug-resistant *M. tuberculosis* in a clinical cohort. By aligning conventional microbiological data with genomic and computational analyses, our findings provide a detailed view of resistance burden, lineage-associated dissemination, and the predictive value of WGS, with relevance to both global and China-specific tuberculosis epidemiology. While several features of tuberculosis resistance have been described previously, this study provides a region-specific, integrative analysis combining phenotypic, genomic, phylogenetic, and machine learning approaches in a high-burden setting of Kashi, where such comprehensive data remain limited.

Our phenotypic analysis revealed a high burden of first-line drug resistance, with more than half of isolates exhibiting resistance to at least one anti-tuberculosis drug and nearly one-fifth classified as MDR. Resistance was dominated by isoniazid, streptomycin, and rifampicin, while resistance to fluoroquinolones and second-line injectable agents remained relatively uncommon. This pattern closely mirrors global tuberculosis resistance trends reported by the WHO, which consistently identify isoniazid resistance as the most prevalent form of drug resistance worldwide, often occurring independently or preceding rifampicin resistance (Goletti et al., 2025). Large-scale global surveillance studies have shown that isoniazid resistance affects approximately 7–10% of new TB cases globally and exceeds 15% in previously treated patients, contributing substantially to treatment failure and progression to MDR-TB (Stagg et al., 2016; Zignol et al., 2016). Our observed resistance frequencies are also highly consistent with national data from China, a country that accounts for one of the largest burdens of drug-resistant TB worldwide. National drug resistance surveys conducted in China have reported MDR-TB prevalence ranging from 5–7% in new cases to 20–25% in previously treated patients, with isoniazid and streptomycin resistance being particularly common (Zhao et al., 2012; Yang et al., 2017a). The relatively low prevalence of XDR isolates in our cohort aligns with more recent reports from China indicating stabilization or modest declines in second-line resistance following expanded access to rapid molecular diagnostics, improved treatment regimens, and strengthened TB control programs (Yao et al., 2021). Together, these comparisons indicate that the phenotypic resistance landscape observed in our study reflects broader global and national resistance patterns rather than a localized anomaly. The relatively high prevalence of streptomycin resistance observed in this study may reflect historical patterns of anti-tuberculosis drug use (Yang et al., 2025). Streptomycin was among the earliest first-line agents widely used in TB treatment, and its extensive historical use may have contributed to the selection and persistence of resistance-associated mutations. In addition, certain streptomycin resistance mutations, particularly in *rps*L and *rrs*, are known to impose relatively low fitness costs, allowing resistant strains to persist and be transmitted within the population. These factors may collectively explain the sustained burden of streptomycin resistance observed in this cohort.

Whole-genome phylogenetic analysis demonstrated that resistant isolates, particularly MDR strains, were not randomly distributed across the population structure, but were enriched within Lineage 2 (Beijing lineage). This finding is highly concordant with extensive global and China-focused genomic studies that have repeatedly linked the Beijing lineage with increased drug resistance and transmission fitness. Multiple population-based studies from China have reported that Lineage 2 strains account for a disproportionately high fraction of MDR-TB cases, particularly in northern and eastern provinces (Luo et al., 2014; Yang et al., 2017b; Merker et al., 2018). Globally, Beijing lineage strains have been associated with MDR-TB outbreaks in East Asia, Russia, Eastern Europe, and parts of Africa, suggesting a lineage-specific capacity for resistance acquisition and clonal expansion (Niemann et al., 2010; Merker et al., 2015; Zhu et al., 2023). Experimental and epidemiological evidence suggests that Lineage 2 strains may tolerate resistance-associated fitness costs more effectively than other lineages, potentially due to compensatory mutations and altered stress response pathways. Our observation that drug-sensitive isolates were distributed across multiple lineages, while MDR isolates clustered within Lineage 2, supports a model in which possible clonal dissemination and shared transmission history, together with lineage-associated evolutionary advantages, contribute to the observed resistance patterns.

Mapping resistance-associated mutations across phenotypic groups revealed a clear, stepwise increase in mutational burden from drug-sensitive to SDR and ultimately MDR isolates. Drug-sensitive isolates were largely devoid of canonical resistance mutations, SDR isolates typically harboured single high-confidence mutations, and MDR isolates accumulated multiple resistance-conferring mutations across distinct drug classes. This progressive mutational pattern is consistent with well-established evolutionary models of tuberculosis drug resistance, in which resistance is acquired sequentially under sustained antimicrobial pressure. Global WGS studies have demonstrated that isoniazid resistance, often driven by *katG* S315T or *inhA* promoter mutations, frequently precedes rifampicin resistance, which is commonly mediated by *rpoB* RRDR substitutions such as S45L (Farhat et al., 2013; Walker et al., 2015). Our findings align closely with these observations, with SDR isolates frequently harbouring single mutations and MDR isolates showing combined alterations in *rpo*B, *kat*G, *emb*B, *gyr*A, and other resistance-associated loci. Chinese genomic studies have reported similar patterns, with MDR isolates exhibiting significantly higher mutational diversity and accumulation of resistance-associated variants compared with drug-sensitive strains (Coscolla et al., 2015; Yang et al., 2017b). The presence of additional mutations in genes such as *pnc*A, *gyr*B, and *rrs* in subsets of MDR isolates further supports the notion of resistance amplification and diversification as treatment pressure intensifies.

Machine learning–based resistance prediction using WGS-derived features achieved high predictive accuracy for rifampicin, isoniazid, and streptomycin resistance, with ROC–AUC values comparable to or exceeding those reported in prior genomic prediction studies. Importantly, feature importance analyses consistently prioritized canonical resistance mutations, providing biological validation of the predictive framework. Previous large-scale studies have demonstrated that WGS-based models can achieve promising predictive performance for resistance to first-line drugs, particularly rifampicin and isoniazid, with AUC values typically ranging from 0.85 to 0.95 (Walker et al., 2015; Miotto et al., 2017). Our findings fall squarely within this range, reinforcing the robustness of WGS-driven prediction approaches. The strong contribution of *rpo*B RRDR mutations to rifampicin prediction and *kat*G S315T to isoniazid prediction is consistent with WHO-endorsed resistance mutation catalogues (Wang et al., 2022). Notably, promoter mutations, particularly in the *inh*A regulatory region, contributed substantially to predictive performance, underscoring the importance of regulatory variation. While WGS-based prediction demonstrated strong performance for major first-line drugs, genomic prediction does not fully capture all mechanisms underlying phenotypic resistance. Resistance phenotypes may be influenced by rare mutations outside currently recognized resistance loci, epistatic interactions among genomic variants, heteroresistance, or regulatory mechanisms not captured by mutation-based approaches alone. Consequently, WGS should be viewed as a complementary tool that augments, rather than completely replaces, phenotypic susceptibility testing, particularly for uncommon resistance phenotypes and disputed mutations. Several global and China-specific studies have highlighted the role of promoter mutations in conferring low-level resistance that may be missed by conventional diagnostics but captured through WGS (Folkvardsen et al., 2013; Miotto et al., 2018). Our results further emphasize that inclusion of non-coding variants enhances both predictive accuracy and biological interpretability. Together, our phenotypic, phylogenetic, genomic, and machine learning analyses converge on a coherent biological narrative that is consistent with global and Chinese tuberculosis epidemiology. The observed resistance burden, lineage-associated clustering, stepwise mutational accumulation, and high predictive performance of WGS-based models collectively support the value of integrated genomic approaches for understanding and monitoring drug-resistant *M. tuberculosis*. The integration of whole-genome sequencing with machine learning–based predictive models has the potential to enhance clinical tuberculosis surveillance by enabling rapid, data-driven prediction of drug resistance and supporting individualized treatment strategies. With further validation in larger and multi-center cohorts, such approaches could be incorporated into routine diagnostic workflows to improve early detection and management of drug-resistant tuberculosis. While the machine learning models demonstrated strong predictive performance, several limitations should be considered. The number of genomic features relative to the sample size (*n* = 160 isolates) raises the potential for overfitting despite the use of cross-validation. In addition, only a Random Forest model was evaluated in the present study. Consequently, it is not possible to determine whether the observed predictive performance is superior, equivalent, or inferior to that achievable using alternative machine learning algorithms (e.g., logistic regression, support vector machines, or gradient boosting methods) or established genomic resistance prediction tools such as TB-Profiler and Mykrobe. Therefore, the present findings should be interpreted as demonstrating the feasibility of WGS-based resistance prediction using a Random Forest framework rather than establishing superiority over alternative machine learning approaches.

While this study provides an integrated phenotypic, genomic, and machine learning–based characterization of drug-resistant *M. tuberculosis*, several aspects warrant consideration and point toward future research opportunities. First, although the cohort size was sufficient to support robust phylogenetic and resistance prediction analyses, inclusion of larger, multi-center datasets encompassing broader geographic regions would further enhance generalizability and enable finer resolution of transmission dynamics. PPV, NPV, and confidence intervals were not calculated because of sample-size limitations and should be evaluated in future larger external validation studies. Future studies incorporating larger datasets and systematic benchmarking are warranted. In addition, detailed genotype–phenotype concordance analyses were not performed in the present study and should be incorporated in future investigations to better characterize discordant resistance patterns and further evaluate the clinical utility of WGS-based resistance prediction. Phenotypic drug susceptibility testing and MIC data were available for major first-line and selected second-line drugs; extension to newer and repurposed agents would allow more comprehensive resistance profiling as treatment strategies evolve. Third, while the machine learning models demonstrated strong predictive performance and biological interpretability, incorporation of additional genomic features such as structural variants, gene expression data, or host clinical variables may further improve prediction accuracy and clinical relevance. Finally, longitudinal sampling and integration of treatment response data would enable direct assessment of resistance evolution over time and facilitate translation of WGS-based prediction frameworks into real-time clinical decision support. Addressing these aspects in future studies will build upon the strengths of the present work and advance the application of genomic and computational approaches for precision tuberculosis control. The very limited number of XDR isolates restricted robust evaluation of predictive performance for highly resistant phenotypes and limited lineage-specific inference for advanced resistance categories. The present machine learning analysis should be interpreted as exploratory due to the relatively limited cohort size and absence of external validation or benchmarking against alternative machine learning algorithms and established genomic resistance prediction tools. Future studies incorporating larger multi-center datasets and comparative modelling approaches will be necessary to establish generalizability and clinical utility. Random Forest was selected due to its suitability for high-dimensional genomic data and its ability to model complex non-linear interactions among resistance-associated mutations. Cross-validation and hyperparameter optimization were implemented to improve robustness and reduce overfitting.

## Conclusion

This study provides a comprehensive and integrated characterization of drug-resistant *M. tuberculosis* by combining phenotypic drug susceptibility testing, whole-genome sequencing–based phylogenetics, resistance mutation profiling, and interpretable machine learning–based prediction. Our findings demonstrate a substantial burden of first-line drug resistance, lineage-associated clustering of multidrug-resistant strains suggesting possible clonal dissemination and shared transmission history, and a clear stepwise accumulation of resistance-conferring mutations underlying phenotypic resistance patterns. Importantly, whole-genome sequencing–derived features enabled accurate and biologically consistent prediction of drug resistance, with both coding and regulatory mutations contributing meaningfully to model performance. Together, these results underscore the value of integrating genomics and machine learning into tuberculosis resistance surveillance frameworks and support the application of genome-informed approaches to enhance precision diagnostics and inform public health strategies for tuberculosis control.

## Data Availability

Raw paired-end FASTQ whole-genome sequencing files generated in this study have been deposited in the National Genomics Data Center (NGDC) (https://ngdc.cncb.ac.cn/gsa/browse/CRA039528), under accession PRJCA058814. Additional de-identified data supporting the findings of this study are available from the corresponding authors upon reasonable request.
