# Supplementary material for: Whole-genome sequencing reveals lineage-associated drug resistance and enables machine learning–based prediction in Mycobacterium tuberculosis clinical isolates
Source: Front Microbiol. 2026 Jul 22;17:1828991. doi: 10.3389/fmicb.2026.1828991 (PMC13437962; doi:10.3389/fmicb.2026.1828991)
Supplement: Supplementary file 1 [file Data_Sheet_1.pdf]

Supplementary File

Whole-Genome Sequencing Reveals Lineage-Associated Drug Resistance and Enables Machine Learning–Based Prediction in *Mycobacterium tuberculosis* Clinical Isolates

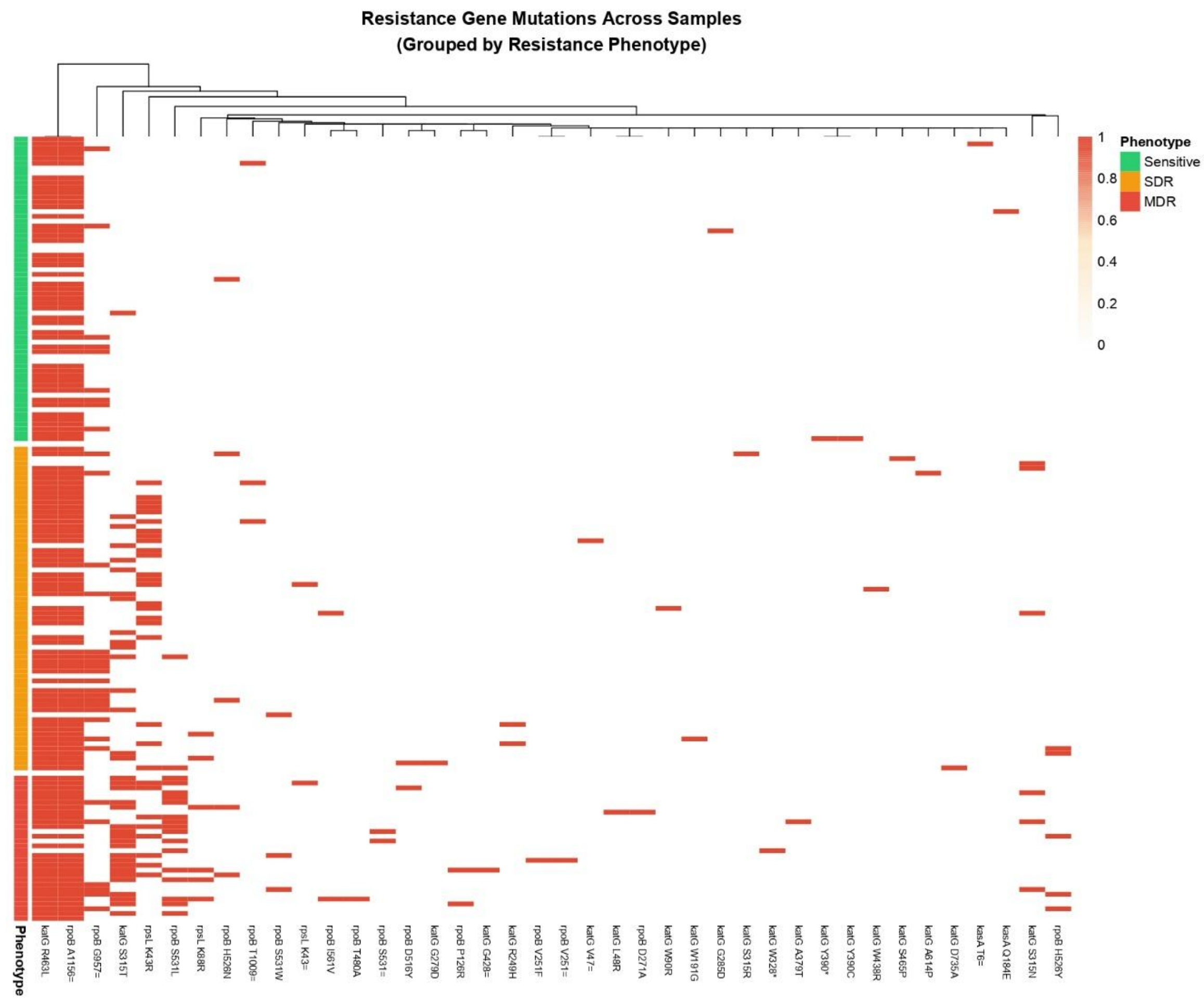

Supplementary Figure S1. Resistance gene mutation landscape stratified by phenotypic drug resistance category.

Comprehensive heatmap depicting resistance-associated mutations across all sequenced *Mycobacterium tuberculosis* isolates, grouped by phenotypic resistance classification (drug-sensitive, SDR, MDR). The figure highlights mutation frequency and distribution across major resistance-associated loci, demonstrating minimal mutation burden in drug-

sensitive isolates, intermediate mutation accumulation in SDR isolates, and extensive multi-gene mutation enrichment in MDR isolates. This supplementary analysis provides high-resolution support for the genotype–phenotype relationships observed in the main analysis.

Supplementary Table S1. Performance metrics of Random Forest models for prediction of drug resistance

Drug	AUC (95% CI)	Sensitivity	Specificity	PPV	NPV	Accuracy
RIF	0.854 (0.774-0.933)	73.7%	91.8%	73.7%	91.8%	87.5%
INH	0.884 (0.822-0.945)	89.1%	80.0%	70.0%	93.3%	83.1%
SM	0.874 (0.807-0.941)	79.6%	89.2%	76.5%	90.8%	86.2%
